# Recommendations for the Regionalizing of Coffee Cultivation in Colombia: A Methodological Proposal Based on Agro-Climatic Indices

**DOI:** 10.1371/journal.pone.0113510

**Published:** 2014-12-01

**Authors:** Juan Carlos García L., Húver Posada-Suárez, Peter Läderach

**Affiliations:** 1 Production and Productivity Program, National Center for Coffee Research (CENICAFÉ), Manizales, Colombia; 2 Agricultural Sciences Faculty, Universidad de Caldas, Manizales, Colombia; 3 Technical Division, National Federation of Coffee Growers of Colombia (FNC), Santafé de Bogotá, Colombia; 4 Decision and Policy Analysis (DAPA), International Center for Tropical Agriculture (CIAT), Cali, Colombia; Tennessee State University, United States of America

## Abstract

The Colombian National Federation of Coffee Growers (FNC) conducted an agro-ecological zoning study based on climate, soil, and terrain of the Colombian coffee-growing regions (CCGR) located in the tropics, between 1° and 11.5° N, in areas of complex topography. To support this study, a climate baseline was constructed at a spatial resolution of 5 km. Twenty-one bioclimatic indicators were drawn from this baseline data and from yield data for different coffee genotypes evaluated under conditions at eight experimental stations (ESs) belonging to the National Center for Coffee Research (CENICAFÉ). Three topographic indicators were obtained from a digital elevation model (DEM). Zoning at a national level resulted in the differentiation of 12 agro-climatic zones. Altitude notably influenced zone differentiation, however other factors such as large air currents, low-pressure atmospheric systems, valleys of the great rivers, and physiography also played an important role. The strategy of zoning according to coffee-growing conditions will enable areas with the greatest potential for the development of coffee cultivation to be identified, criteria for future research to be generated, and the level of technology implementation to be assessed.

## Introduction

Coffee is one of the most important commodities in the international agricultural market and a source of income for many countries in Asia, Africa and Latin America. In the period from 1965 to 1995, Colombia contributed to an average of 13.5% of world production, and between 2000 and 2011 to 7.6% [1]. The coffee crop (*Coffea arabica*) represents 17% of Colombia's agricultural gross domestic product and constitutes 9% of its agricultural output. About 2.2 million people depend directly on coffee for their livelihoods, this figure is equivalent to 25% of Colombia's rural population and 31% of its national labour force employed in agriculture [2]. Much of this employment is seasonal, part-time and informal [1], with jobs directly generated by the coffee industry distributed among the following activities: investment (3.9%); management (65.2%); harvest (29.5%); and postharvest (1.4%) [3].

The Colombian coffee-growing regions, lie between 1° and 11.5° N, and 72° and 78° E, encompassing the Western, Central, and Eastern Andean Ranges, as well as the mountain system of the Sierra Nevada of Santa Marta in northern Colombia [4]. Coffee plantations are found at altitudes between 800 and 2000 masl.

CENICAFE has experimental stations (ESs) located in important coffee-growing areas, in the states of Caldas, Antioquia, Tolima, Risaralda, Cauca, Cundinamarca, Cesar and Quindío. These highly technological coffee farms include the Central Experimental Station Naranjal, ES Rosario, ES La Trinidad, ES La Catalina, ES El Tambo, ES Santa Bárbara, ES Pueblo Bello and ES Paraguaicito.

In Colombia, the intertropical convergence zone is responsible for the existence of two dry and two wet seasons per year [4], [5], [6]. These seasons determine the two coffee harvesting periods, with variations in the northern and southern extremes of the CCGR where a mono-modal rainfall distribution results in a concentrated harvest [4], [5], [6], [7]. The relative intensity of the dry season (1 to 2 months) has repercussions on the production cycle, from flowering to harvesting, with variability observed between 215 to 240 days at 5° N and 11° N, respectively [5].

Colombia is characterized by climatic complexity, with temporal variability rendering the association of a pattern of reaction to an agronomic variable with given climatic elements, as difficult. The country's climate was first classified by Hurtado into seven groups using Thornthwaite's classification criteria [8]. Later, Baldión and Hurtado [9] proposed five groups based on agro-climatic indices derived from hydric balances obtained through Palmer's method [10] which collected climate information over a period of 10 years. More recently, Malagón et al. introduced the concept of bioclimatic factors related to soil formation, emphasizing the importance of temperature and soil moisture in soil evolution [11].

The FNC studied soils, climates, and terrains in the coffee-growing regions defined by the 1980–1981 Coffee Plantations Census. In total, 86 agro-ecological zones known as *ecotopes* were identified where coffee trees responded to their environment in similar ways and where geographic area was homogenous and continuous [4].

In several studies in Brazil, the use of indicators for coffee has permitted the following activities:

- Estimation of the length of different phenological periods [12], [13], [14]
- Development of agro-climatic models for estimating productivity [15], [16]
- Construction of agro-climatic zones for delimiting homogeneous areas by their performance and defining their limitations, advantages, and risks [17], [18]
- Design of frost-alert systems [19]

In Colombia, indices have been constructed taking into account the crop's physiological periods, in particular, flowering [20], [21], fruit development [7], and the entire cycle from planting to harvest [22]. These indices help to establish criteria for season planning [23], [24], [25].

This research aims to identify coffee-growing areas with similar agro-climatic characteristics and determine if the scope of current research is sufficiently regional in terms of its coverage. This will contribute to important future decision-making processes by coffee growers in the diverse regions of the country.

## Materials and Methods

The methodology consisted of defining and acquiring the baseline and the bioclimatic indicators, and then incorporating field attributes of the coffee-growing regions. This methodology was adopted following previous analysis which used climatic elements such as annual precipitation and temperature. The results of the agro-climatic groups (ACGs) obtained are presented in a later section of this paper.

### 2.1. Physiological data

#### 2.1.1. Information on harvesting patterns

Based on Arcila et al. [23], a harvest raster adjusted to the Colombian coffee-growing regions was generated using two criteria: the main harvest predominating in the first semester (between January and June) and the main harvest predominating in the second semester (between July and December). These criteria were used to construct the coffee tree's physiological stages (detailed below), with their corresponding peak harvesting months for the zones with first and second semester harvests (May and October, respectively).

#### 2.1.2. Consolidation periods and physiological phases

Three physiological phases were defined as occurring before the main harvest, relating to the bioclimatic indices described above:

- *Four months before maximum flowering* (which defines the principal harvest): hereafter referred to as stage 1. This phase begins with the flowering induction, followed by the appearance of latent floral buds, and finally the occurrence of flowering after a rainfall. [20], [26].
- *First four months of berry development* (towards the principal harvest): hereafter referred to as stage 2. In this phase, the completion of the early phases of coffee berries development towards final seed size take place. [7], [26].
- *Four months before the principal harvest*: hereafter referred to as stage 3. In this phase coffee berries acquire their uniformity and final weight. [7], [26].

### 2.2. Environmental data

#### 2.2.1. Climate information

More than 20 years of historical information on precipitation, temperatures (minimum, mean, and maximum), and solar brightness from 80 meteorological stations of the FNC's coffee climate network was used for this study. Daily information from the coffee-growing regions was modelled using Hutchinson's methodology [27] together with the ANUSPLIN interpolator, version 4.3 (which uses geographic coordinates and terrain elevation as independent variables). This procedure has been used in global studies undertaken by Hutchinson [28] and others [29], [30], [31], [32], [33], [34]. Usually, the strategy of generating daily data requires the adaptation of programming routines in the R Platform [35], [36].

#### 2.2.2. Information on the water retention capacity of soil

Soil water retention (*SWR*), also known as maximum storage in hydric balance, is defined in terms of field capacity (fc), permanent wilting point (pwp), apparent density (*ad*), and depth of the coffee tree's root zone (*d*). The formula is as follows:


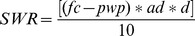
 [37]

Information on the shape of soil units (digitized from findings in FNC's framework study on coffee *ecotopes* [4]) was crossed with the results of the physical characterization (fc, pwp, *ad*, *d*) carried out by Suárez [38] on some of these units. A raster with information on soil water retention was generated. To assure the zone's continuity, in areas not covered by Suárez's study [38] a theoretical daily retention capacity of 50 mm was assigned, based on test results from hydric balances of CENICAFÉ's Agroclimatology Research Group.

#### 2.2.3. Generating buffer zones adjusted to CCGR

Following the delimitation of coffee-growing plantations or farms, additional bordering areas or buffer zones of 3 km wide were generated to cover the edges of coffee-growing regions and facilitate generation of daily information on bioclimatic indices. Through this information, 5789 pixels or centroids across CCGR were obtained.

#### 2.2.4. Constructing the bioclimatic indices

Twenty one bioclimatic indices were obtained and classified into 3 groups: 9 moisture indices, 6 solar brightness indices and 6 thermal indices. Most bioclimatic indicators were developed on a point basis, given that they were associated with, for example, meteorological stations collecting largely pluviometric information together with historical information.

**Moisture indices**: To calculate the daily hydric balance, a routine was generated in R Platform [35], according to the methodology described and adapted by Jaramillo et al. [39], [37] At the end of the routine, the soil water index (SWI) was obtained (i.e. the difference between real evapotranspiration [ET_r_] and potential evapotranspiration [ET_p_]). Its values are expressed between 0 and 1, where 0 corresponds to completely dry soil, and 1 to all the porous spaces being filled. Moderate hydric deficit (MHD) falls in the range 0.5≤ SWI ≤0.8, while severe hydric deficit (SHD) is established at SWI <0.5. For each stage, the number of days, and the accumulated daily rainfall (ppt) observed satisfied the criteria for one of the two indices. The following bioclimatic indicators were generated:

ppt1  =  accumulated rainfall, stage 1
ppt2  =  accumulated rainfall, stage 2
ppt3  =  accumulated rainfall, stage 3
md1  =  number of days with MHD, stage 1
md2  =  number of days with MHD, stage 2
md3  =  number of days with MHD, stage 3
sd1  =  number of days with SHD, stage 1
sd2  =  number of days with SHD, stage 2
sd3  =  number of days with SHD, stage 3

**Solar brightness indices**: An R Platform routine was generated to calculate solar radiation (SR), using Campbell and Donatelli's methodology as described by Meza and Varas [40] and Rivington et al. [41], [42]. Solar brightness (SB) is calculated from SR, based on (a) coefficients a and b obtained by Gómez and Guzmán [43], using the Ångström formula, and (b) the methodology presented in Appendix C of the *Atlas de Radiación Solar de Colombia* [44]. The difference between the duration of the astronomical day in hours and SB gives the solar brightness deficit (SBD). For each of the physiological stages established, the hours of SB were counted, together with days where SBD was <7.2 [21], to generate the following bioclimatic indicators:

sb1  =  accumulated SB, stage 1
sb2  =  accumulated SB, stage 2
sb3  =  accumulated SB, stage 3
bd1  =  number of days with SBD at <7.2, stage 1
bd2  =  number of days with SBD at <7.2, stage 2
bd3  =  number of days with SBD at <7.2, stage 3

**Thermal indices**: The indices for Thermal Amplitude (TA) or thermal gradient (*T_max_ – T_min_*,) and Thermal Time (TT) or degree days (*T_mean_ – T_base_*) were generated from information on maximum (*T_max_*), minimum (*T_min_*), and mean (*T_mean_*) temperatures, and with the lowest base temperature (*T_base_*) of 10°C, as determined for coffee trees in Colombia by Jaramillo and Guzmán [22]. For each of the three physiological stages proposed, the TT and the number of days with TA at<10 were accumulated [21]. The following bioclimatic indices were generated:

tt1  =  accumulated TT, stage 1
tt2  =  accumulated TT, stage 2
tt3  =  accumulated TT, stage 3
ta1  =  number of days with TA at <10, stage 1
ta2  =  number of days with TA at <10, stage 2
ta3  =  number of days with TA at <10, stage 3

#### 2.2.5. Incorporating the bioclimatic indices to the geo data base

As well as constructing the 21 bioclimatic indices, each of the 5789 centroids was associated with the physiographic components of aspect, shade, and slope, thus incorporating 24 attributes per pixel. This also served to geo-reference the pixels.

#### 2.2.6. Topographic information

Terrain attributes such as elevation, slope, hillside shade, and aspect were generated from the DEM of the Shuttle Radar Topography Mission [45]. A resolution of 5 km was used for national zoning, taking into consideration only pixels where the area covered by coffee was more than 30%.

### 2.3. Statistical methodology

#### 2.3.1. Multivariate analysis

The multivariate analysis described by Peña and Díaz [46], [47], and the statistical package “ade4” [48] in the R platform were used. The selection of synthetic variables was based on the maximum degree of variability that was explained by the PCA, where the eigenvalues were equal to or greater than 1. Due to the fact that the original variables were standardized before the PCA was performed, the means of the standardized variables were zero and the variances were equal to one.

A cluster analysis was also undertaken, using PCAs from the previous analysis. Two aspects were considered: similarity measures and clustering methods [46], [47]. For the first aspect, according to the method, the proximity of observations must be measured; in this case, the Euclidean distance was used. For the second aspect, clusters were formed, whereby observations were selected to be as similar and as different as possible within and between clusters, respectively. K-means clustering, a partitioning method that assumes the existence of an Euclidean distance between the members comprising the cluster, was used to construct this time series [49], [50]. Indices of similarity and quality as proposed by Liao [49] were assumed as criteria for evaluating and deciding on cluster formation. The R routine was adapted to the needs of the current research, using the statistical package “cclust” from R Platform [51].

## Results

### 3.1. Forming agroclimatic groups for the CCGR

Six principal components represented 86% of the variability attributable to the original 24 variables (21 bioclimatic and 4 topographic indices). The first component explained 34% of total variation, comprising most of the bioclimatic indicators; except sd2, sb2, ppt1, ta3, sb3, bd3, md1, and sd1, which were not significant. The second component explained 21.5% of the variation and was composed of six bioclimatic indicators: sb2, sb3, bd3, ta3, ppt1, and sd1. Components 3 to 6 explained 11.7, 7.5, 6.6, and 5.0% of the variation respectively. Component 5 was represented by the topographic indicators of aspect and shade. *Slope* showed a relationship with component 6 (Table 1).

**Table 1:** Principal Component Analysis from the twenty four bioclimatic indices.

Principal Component	Eigenvalues	Explication of the Variability
1	8.13	33.90%
2	5.15	55.40%
3	2.81	67.10%
4	1.81	74.60%
5	1.58	81.20%
6	1.2	86.10%

**Figure 1:**
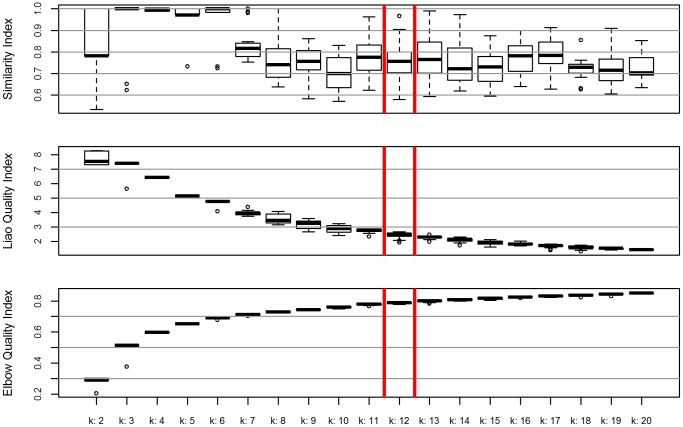
Boxplot from three indexes, Quality of Elbow and Similarity and Quality of Liao, built to determine the best decision criteria for groups, in an analysis of k-means clustering in the ACG. The axis "x" represents the k group level and the axis “y" the value of each index, the first and last values are expressed from 0–1, with 1 being the perfect fit. The red box highlights the group with best fit.

The six components were taken into account in the cluster analysis. The clustering test considered 40 combinations for 39 possible groups with 100 iterative processes for each one. The cluster for agroclimatic group 12 (ACG 12) showed three situations of interest: (a) a similarity index mean value of 75% and the least fluctuation on the range of all the groups, even though the extreme values were 64 and 90%; (b) a quality index mean value of 2.47 with minimum variation; and, (c) 78.9% of variability explained, with a fluctuation between 77.5 and 79.5% (Figure 1).

The above-mentioned results show the need to subject the indices to increased control when deciding on the number of groups to be formed.

The process focused on seeking, within each of the 12 ACGs, the particular conditions that differentiated them. Table 2 lists, for each ACG, the mean values of the 21 bioclimatic and 4 topographic indices (including altitude obtained from a DEM with a resolution of 90 m).

**Table 2:** Mean values that discriminate, using 21 bioclimatic and 4 topographic indices, among 12 agro-climatic groups (ACGs) resulting from cluster analysis for the Colombian coffee-growing regions.

ACG	Bioclimatic Indicator																					Topographic Indicator			
	sb1	sb2	sb3	sd1	sd2	sd3	md1	md2	md3	bd1	bd2	bd3	ta1	ta2	ta3	pp1	pp2	pp3	tr1	tr2	tr3	hs	asp	slp	elev
**1**	510	626	575	1	0	0	43	14	0	54	53	42	43	72	66	537	868	886	1194	1236	1163	175	124	4.66	1698
**2**	598	482	588	0	0	0	11	1	3	55	30	63	33	59	42	598	772	771	916	982	967	181	167	4.81	1824
**3**	575	656	526	0	0	0	25	6	0	78	57	20	21	37	53	597	1048	1116	1039	1046	897	176	135	4.93	1815
**4**	667	363	431	96	1	0	22	16	34	75	2	0	61	123	120	304	749	674	1327	1288	1288	185	279	4.14	1512
**5**	585	708	715	5	0	0	46	14	2	84	111	104	14	15	7	506	832	820	1135	1196	1131	183	223	3.00	1660
**6**	666	732	643	51	29	0	24	18	18	92	103	73	16	28	34	398	729	1033	1299	1450	1437	187	254	5.48	1207
**7**	483	627	644	1	0	0	48	28	45	34	59	77	67	95	52	561	771	714	1284	1343	1329	179	176	4.02	1536
**8**	244	420	636	1	23	81	12	31	15	0	0	75	120	122	58	726	603	395	1260	1314	1363	177	140	3.30	1410
**9**	378	544	619	1	1	19	38	29	39	0	6	68	120	122	89	660	782	689	1368	1447	1484	176	128	2.99	1362
**10**	390	569	712	7	20	45	43	41	29	0	46	101	119	107	30	623	673	668	1375	1502	1567	188	277	4.27	1187
**11**	387	517	643	1	7	54	24	31	33	0	2	84	118	122	45	622	650	476	1122	1142	1119	178	121	3.36	1646
**12**	688	452	562	51	1	0	56	16	28	92	12	40	43	105	94	398	702	675	1135	1158	1174	184	277	3.33	1715

a This symbol and the next within the same row, refer to indices, where sb1  =  accumulated solar brightness (SB), stage 1; sb2  =  accumulated SB, stage 2; sb3  =  accumulated SB, stage 3; sd1  =  number of days with severe hydric deficit (SHD), stage 1; sd2  =  number of days with SHD, stage 2; sd3  =  number of days with SHD stage 3; md1  =  number of days with moderate hydric deficit (MHD), stage 1; md2  =  number of days with MHD, stage 2; md3  =  number of days with MHD, stage 3; bd1  =  number of days with solar brightness deficit (SBD) at <7.2, stage 1; bd2  =  number of days with SBD at <7.2, stage 2; bd3  =  number of days with SBD at <7.2, stage 3; ta1  =  number of days with thermal amplitude (TA) at <10, stage 1; ta2  =  number of days with TA at <10, stage 2; ta3  =  number of days with TA at <10, stage 3; ppt1  =  accumulated rainfall, stage 1; ppt2  =  accumulated rainfall, stage 2; ppt3  =  accumulated rainfall, stage 3; tt1  =  accumulated thermal time (TT), stage 1; tt2  =  accumulated TT, stage 2; tt3  =  accumulated TT, stage 3; hs  =  hillshade; asp  =  aspect; slp  =  slope; and elev  =  elevation.

#### 3.1.1. Distribution of experimental stations and the coffee climate network in the setting of agro-climatic groups

**Figure 2:**
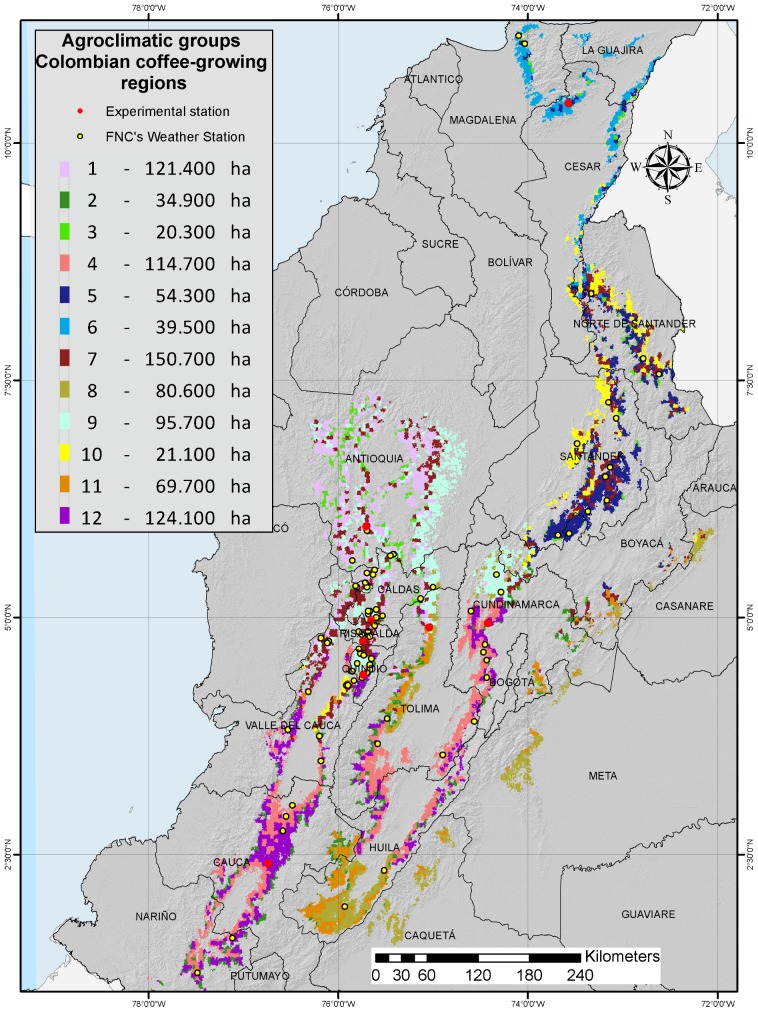
Agroclimatic groups across Colombian coffee-growing regions.

The red dots in Figure 2 show the distribution of CENICAFÉ's ESs throughout the ACGs. Four ESs — El Rosario, Naranjal, La Trinidad, and La Catalina — lie within ACG 9, whereas ESs El Tambo and Santa Bárbara lie within ACG 12. The two remaining ESs are situated in different ACGs, namely, ES Pueblo Bello in ACG 6 and ES Paraguaicito in ACG 4. The main stations in the coffee climate network, totaling 74 and forming part of CENICAFÉ's ESs, are represented in Figure 2 by yellow dots. Aside from ACG 2, they are distributed throughout all the ACGs, cover different types of areas.

#### 3.1.2. Description of the agro-climatic groups

Tables 2 and 3 characterize the ACGs, showing bioclimatic and topographic differences, and other characteristics such as varieties and luminosity. The last column of Table 3 provides the ranges of the most noteworthy bioclimatic and topographic indicators. In particular, the ACGs present variable ranges of altitude, from the predominantly low as in ACGs 6 and 10, in which sd1 is accentuated with more than 59% of its coffee-growing area under shade, to ACGs found mostly in high zones (ACGs 2, 3, and 12), where thermal time values between flowering and harvest are predominantly less than 2500 hours (Figure 2).

**Table 3:** Characteristics associated with the groups that conform the agro-climatic zones proposed for the Colombian coffee-growing regions.

Agro-climatic zone or group (ACG)	Coffee area and lands	Departments and representation within the ACG		Proportion by Andean range within the ACG			Proportion by latitudinal zone and luminosity within the ACG		Proportion by variety within the ACG		Bioclimatic indicators (range for 80% of coffee farms)	
		Dep′t	Propor. (%)	Range	Flank	Propor. (%)	Latitudinal zone/Luminosity	Propor. (%)	Variety	Propor. (%)	Indicator	Range
1	121,400 ha	Antioquia	58	Occidental (*Western*)	East	52	Central-northern	80.4	Caturra	44.8	Altitude (masl)	1400–1940
	92,900 farms	Caldas	14.4	Central	West	7.7	Central-southern	15.7	Colombia	33.6	Solar brightness (h/yr)	1660–1760
		Risaralda	10.1		West	20.8			Castillo	18.3	Annual rainfall (mm)	2110–2470
		Valle del Cauca	8.2		East	19	Sun	68.2	Typica	3.2	MHD, stage 1 (days)	32–46
		Tolima	7.7				Semi-shade	22.9			TA, stage 1 (days)	30–60
							Shade	8.8			TT (accumulated, stages 2 and 3)	2150–2650
2	34,000 ha	Tolima	30.6	Central	East	46.2	Central-southern	52.9	Caturra	53.4	Altitude (masl)	1600–2050
	32,700 farms	Cauca	16.6		West	30.2	Southern	41.1	Castillo	19.5	Solar brightness (h/yr)	1620–1750
		Huila	15.9	Oriental (Eastern)	West	13.8	Central-northern	6.1	Colombia	16.7	Annual rainfall (mm)	2010–2400
		Nariño	12.4		East	2			Typica	10.3	MHD, stage 1 (days)	<32
		Cundinamarca	8.6	Occidental	East	5.7	Sun	60.2			TA, stage 1 (days)	18–52
		Valle del Cauca	6		West	1.1	Semi-shade	30.2			TT (accumulated, stages 2 and 3)	1730–2080
							Shade	9.6				
3	20,300 ha	Antioquia	51.7	Central	West	24.2	Central-northern	72.2	Caturra	50.9	Altitude (masl)	1540–2060
	19,200 farms	Caldas	13.5		East	14.1	Northern	26	Colombia	20.5	Solar brightness (h/yr)	1700–1900
		Cesar	10.7	Occidental	East	30.6			Castillo	18.7	Annual rainfall (mm)	2570–3100
		Risaralda	5.7		West	5.7	Sun	59.4	Typica	9.8	MHD, stage 1 (days)	11–35
		Norte de Santander	5.1	Oriental	West	12.7	Semi-shade	28.2			TA, stage 1 (days)	10–28
		Magdalena	4.4		East	5.2	Shade	12.4			TT (accumulated, stages 2 and 3)	1680–2160
		Santander	3.3	Sierra Nevada		6.8						
		Tolima	3									
4	114,700 ha	Tolima	26.2	Central	West	33.6	Central-southern	56.4	Caturra	35.8	Altitude (masl)	1200–1780
	105,200 farms	Cauca	22		East	23.1	Southern	42	Colombia	29	Solar brightness (h/yr)	1160–1590
		Cundinamarca	16	Oriental	West	34			Castillo	20.6	Annual rainfall (mm)	1600–1920
		Huila	14.8	Occidental	East	7.1	Semi-shade	44.9	Typica	14.6	SHD, stage 1 (days)	80–100
		Nariño	11.8		West	2.1	Sun	39.8			TA, stage 1 (days)	33–120
		Valle del Cauca	6.6				Shade	15.3			TT (accumulated, stages 2 and 3)	2440–2800
5	54,300 ha	Santander	48.6	Oriental	West	67.9	Central-northern	55.3	Colombia	34.4	Altitude (masl)	1370–1880
	41,600 farms	Norte de Santander	25		East	23.5	Northern	44.7	Caturra	28.1	Solar brightness (h/yr)	1940–2060
		Boyacá	10.5	Sierra Nevada		8.3			Typica	21.1	Annual rainfall (mm)	2020–2400
		Cesar	9.7				Semi-shade	52	Castillo	16.4	MHD, stage 1 (days)	32–57
		Magdalena	3.3				Shade	38.2			TT (accumulated, stages 2 and 3)	2100–2490
		La Guajira	2.8				Sun	9.7				
6	39,500 ha	Cesar	43.9	Sierra Nevada		63.2	Northern	100	Typica	43.9	Altitude (masl)	840–1600
	10,900 farms	Magdalena	42.9	Oriental	West	34.8			Caturra	28.8	Solar brightness (h/yr)	1980–2210
		La Guajira	10.2		East	1.5	Semi-shade	63	Castillo	14.2	Annual rainfall (mm)	2020–2400
		Norte de Santander	2.9				Shade	29.4	Colombia	12.9	MHD, stage 1 (days)	19–29
							Sun	7.5			SHD, stage 1 (days)	39–59
											TT (accumulated, stages 2 and 3)	2000–2330
7	150,700 ha	Risaralda	20.9	Occidental	East	34.5	Central-northern	55.1	Colombia	40.4	Altitude (masl)	1270–1800
	85,000 farms	Caldas	19.7		West	7.4	Central-southern	36.6	Caturra	34.5	Solar brightness (h/yr)	1670–1870
		Valle del Cauca	19.6	Central	West	32.7	Northern	8.3	Castillo	18.2	Annual rainfall (mm)	1940–2200
		Antioquia	17.9		East	8.8			Typica	6.9	MHD, stage 1 (days)	42–55
		Santander	7.9	Oriental	West	9.5	Sun	56.9			SHD, stage 3 (days)	26–57
		Norte de Santander	6.3		East	7.2	Semi-shade	29.9			TT (accumulated, stages 2 and 3)	2480–2870
		Tolima	2.7				Shade	13.1				
		Quindío	2.5									
		Boyacá	1.1									
8	80,600 ha	Huila	70.9	Oriental	West	56.6	Southern	76.5	Caturra	59.6	Altitude (masl)	1110–1680
	59,900 farms	Tolima	15.8		East	9.2	Central-southern	18.6	Castillo	18.4	Solar brightness (h/yr)	1640–2080
		Caquetá	3.5	Central	East	33.7	Central-northern	4.9	Colombia	18.2	Annual rainfall (mm)	1140–1400
		Meta	3.1						Typica	3.7	MHD, stage 2 (days)	20–40
		Casanare	2.4				Sun	76			SHD, stage 3 (days)	60–80
		Cauca	2.1				Semi-shade	19			TT (accumulated, stages 2 and 3)	2430–3050
							Shade	5				
9	95,700 ha	Caldas	29.8	Central	West	34.5	Central-northern	69.6	Colombia	40	Altitude (masl)	1080–1660
	63,700 farms	Antioquia	24.9		East	33.2	Central-southern	29.1	Caturra	38.6	Solar brightness (h/yr)	1360–1660
		Tolima	14	Occidental	East	21.8	Northern	1.3	Castillo	18.8	Annual rainfall (mm)	2010–2300
		Quindío	9.7		West	3			Typica	2.6	MHD, stage 1 (days)	26–46
		Risaralda	8.2	Oriental	West	7.4	Sun	69.6			MHD, stage 3 (days)	31–51
		Cundinamarca	6.2				Semi-shade	22			TT (accumulated, stages 2 and 3)	2690–3210
		Valle del Cauca	6				Shade	8.3				
10	21,100 ha	Norte de Santander	35.3	Oriental	West	42.4	Northern	53.4	Colombia	36.5	Altitude (masl)	820–1520
	11,800 farms	Santander	30		East	33.5	Central-southern	23.9	Caturra	28.7	Solar brightness (h/yr)	1510–1770
		Valle del Cauca	23.9	Central	West	23.6	Central-northern	22.7	Typica	20.2	Annual rainfall (mm)	1830–2140
		Cesar	7.1						Castillo	14.6	MHD, stage 1 (days)	33–53
							Semi-shade	59			SHD, stage 3 (days)	26–56
							Shade	24.6			TT (accumulated, stages 2 and 3)	2830–3390
							Sun	16.4				
11	69,700 ha	Huila	60.6	Central	East	58.9	Southern	66.8	Caturra	60.6	Altitude (masl)	1360–1860
	52,000 farms	Tolima	24.2		West	3.1	Central-southern	30	Castillo	18.9	Solar brightness (h/yr)	1450–1660
		Cauca	5.4	Oriental	West	31.2	Central-northern	3.2	Colombia	15.5	Annual rainfall (mm)	1640–1900
		Valle del Cauca	4.9		East	4.8			Typica	5	MHD, stage 3 (days)	19–62
		Boyacá	2.3	Occidental	West	1.3	Sun	76			SHD, stage 3 (days)	14–75
					East	0.5	Semi-shade	18.6			TT (accumulated, stages 2 and 3)	2060–2580
							Shade	5.3				
12	124,100 ha	Cauca	36	Central	West	56.5	Southern	55.2	Caturra	49.8	Altitude (masl)	1490–1920
	130,600 farms	Nariño	14.3		East	13.2	Central-southern	41.9	Castillo	20.1	Solar brightness (h/yr)	1590–1750
		Tolima	10.8	Oriental	West	16.8	Central-northern	2.8	Colombia	19.9	Annual rainfall (mm)	1690–1900
		Cundinamarca	10.7	Occidental	East	10.1			Typica	10.2	MHD, stage 1 (days)	38–76
		Quindío	10.6		West	3.3	Semi-shade	43.6			MHD, stage 3 (days)	14–39
		Valle del Cauca	9.2				Sun	43.2			SHD, stage 1 (days)	8–75
		Huila	8.6				Shade	13.2			TT (accumulated, stages 2 and 3)	2140–2470

## Discussion

### 4.1. Agro-climatic groups

The cluster analysis describes relevant characteristics that either contribute to, or limit coffee production. The methodology is based on factors that occur before the crop's principal harvest, over the three stages of the reproductive period, that is, the physiological events of pre-flowering, flowering, and fruit growth until harvest. Seasonal analysis is determined through the way in which the baseline is obtained - daily history for an average year - whereby the goal is to analyze the performance of the climatic indices.

Table 4 presents advantages and disadvantages of the ACGs according to their agro-ecological suitability for the coffee crop in Colombia. This information is based on agro-climatic indices values drawn from the literature and based on research on the coffee crop in Colombia and Brazil.

**Table 4:** Description of suitability of agroclimatic zones proposed for the Colombian coffee-growing regions.

Agroclimatic zone	Limitations	Advantages	Recommendations
1 and 4	-Slow vegetative and reproductive growth in high areas.	-Zones are suitable for the crop.	-Management with mulch.
		-Flowering tends to be concentrated in two periods.	-High planting densities and arranged in wide alleys.
		-Longer renovation cycles.	-Planting at the beginning of the rainy season.
2 and 3	-Zones are affected by the La Niña phenomenon.	-Zones can become suitable for cultivation under conditions of the El Niño phenomenon.	-Management with mulch and semishade.
	-Excess humidity does not permit concentration of flowering.		-Medium planting densities and arranged in wide alleys.
	-Risk of diseases such as rots caused by Phoma spp., especially at higher altitudes.		-Planting at the beginning of the rainy season.
	-Slow vegetative and reproductive growth.		
5 and 6	-In both zones, shaded conditions may limit production.	-Concentrated flowering and harvesting times.	-Planting at the beginning of the rainy season.
	-Risk of hydric deficit in the middle phase of fruit development in zone 6.	-Longer renovation cycles.	-Regulating shading so that it is no more than 50%.
	-Slow vegetative and reproductive growth at higher altitudes, principally in zone 5.		-Conservation practices with mulching in the dry season.
7, 8, and 9	-Risk of hydric deficit in the late phases of fruit development.	-Flowering frequently concentrates into one semester.	-Management with mulch or transitory shading that favor humidity in stage 3.
	-These zones can lose their suitability for coffee cultivation under conditions of the El Niño phenomenon.	-Sufficient thermal availability.	-Planting at the beginning of the two rainy seasons.
	-Shorter renovation cycles.	-Optimal distribution in coffee border lands.	
10	-Cropping in agroforestal systems because of the temporariness of rainy seasons.	-Flowering frequently concentrates into one semester.	-Management with mulch to favor humidity in stages 2 and 3.
	-This zone can lose its suitability for cultivation during conditions of the El Niño phenomenon.		-Regulating shading so that it is no more than 60%
	-Shade can diminish thermal availability.		-Medium to high planting densities and arranged in wide alleys.
	-Shady conditions can limit production.		-Planting at the beginning of the rainy season.
11 and 12	-Slow vegetative and reproductive growth.	-Flowering frequently concentrates into one semester.	-Medium to high planting densities and arranged in wide alleys.
	-Risk of hydric deficit in the late phases of fruit development.	-Longer renovation cycles.	-Regulating shading so that it is no more than 45%.
	-Zones may lose suitability for cropping under conditions of the El Niño phenomenon.		-Management with mulch to favor humidity in stage 3.
	-Thermal availability diminishes under cloudy conditions.		
	-Risk of diseases such as rots caused by Phoma spp.		

In general, planting time dates determines crop development. At high elevations, the reproductive stage is reached later than at lower altitudes. In some ACGs, hydric deficit during the last phases of fruit development could be improved by adopting management practices such as mulching and establishing live barriers on steep hillsides [52], [53]. In other ACGs, high humidity prevailing throughout most of the crop's reproductive development may favour the appearance of diseases such as those caused by *Phoma* sp. (dieback) and *Erithricium salmonicolor* (pink disease). During flowering, star flower or other abnormalities and attacks from fungi such as *Colletotrichum* sp. (anthracnose) may also appear [52], [54], [55], [56].

As growing coffee under shade may also limit yield [57], practices through the dry period such as regulating shade, sanitary harvesting, and pruning the crop, reduce the potential effects of pests and diseases [58], [59]. Agronomic management of the crop, such as fertilizer application, weed control, mulching, and shade management, reinforces the conditions for a suitable crop [58], [59], [60], [61].

### 4.2. General considerations on agro-climatic group formation

In Colombian coffee cultivation, the concept of latitudinal zoning has been used in agronomical management. In this context, such differentiation results in at least four zones, which are related to flowering patterns [5], [23], [62], [63]: (a) southern zone, delimited between 1° and 3° north; (b) central-southern zone, between 3° and 4° north [5] and 4° in the west, 5° in the north, and 6° in the east; (c) central-northern zone, between 5° and 8° north; and (d) northern zone, between 9° and 11° north.

As indicated above in the description of ACG formation, altitude exerts a strong influence on agro-ecological suitability of areas for coffee cultivation. The four latitudinal zones are associated with the ACGs as follows: the northern zone with ACGs 5, 6, and 10; the southern zone with ACGs 4, 11, and 12; the central-southern zone (the piedmont of the plains and south of Huila) with ACG 8; and the central-northern zone with ACGs 1, 7, and 9. For the northern, southern, and central-southern zones, these associations with the ACGs clearly delineate the influence of the great northeastern air currents and the atmospheric systems of the Pacific Ocean and the Amazon Basin, respectively [6], [64]. The broad valleys forming the Magdalena River's central watershed and the Cauca River watershed noticeably influence the formation of ACGs 1, 7, and 9. Only ACGs 2 and 3 are primarily governed by altitude, which averages at 1800 m above sea level.

These findings present a dimension beyond the geographic, orographic concept or historical development when involving the level of detail such as water retention, solar brightness, degree days, and certain topographic conditions. These aspects brought together, delimit the crop agro-climatically, defining its potential.

Depending on the extent to which information is available for association with a given farm or region, future work will approximate the concept of site-specific agriculture, similar to what was developed for Colombia by CENICAÑA [65], [66], integrating environmental concepts with management concepts. Pilot studies for coffee such as those undertaken by Cock et al. [66], Läderach et al. [67] and Oberthür et al. [68] to obtain the “denomination of origin” for Nariño and Cauca, will determine the future for coffee growers and the FNC, safeguarding farmers from variability in terms of both climate and prices, and enabling progress to be made towards guaranteeing a quality product.

## Recommendations

Spatial resolution at 5 km used to obtain the indices is limited, especially for climatic elements such as precipitation and for topographical features such as slope and altitude. In steep zones, where slopes are more than 25°, the changes associated with altitude, precipitation, and solar radiation within a cell of 5 km are large. Assuming only one class for each element will consequently distort these extreme conditions. The advantages of using this resolution are (a) an association of large surfaces in a continuous manner incorporating data into each cell; (b) efficient use of hardware and software resources; and (c) improved level of precision of information generated.

Although the objective of establishing the potential scope of research results generated by the ESs was achieved, the level of dispersion of the coffee climate network did not allow a higher level of precision. An option to consider is to incorporate more historical series type of information from weather stations, both within and outside the coffee-growing regions, as administered by national agencies such as the Institute of Hydrology, Meteorology, and Environmental Studies (IDEAM) or private companies such as sugar mills. This would result in benefits in terms of consistency of information, the possibility of increasing the level of resolution and therefore the level of detail, and the possibility of exploring other methodologies based on functional geo-statistics, functional regression, and other tools of interpolation to obtain a greater coverage with improved level of confidence.

One factor that limited the process of obtaining bioclimatic indicators was the restricted scope of soil studies. Another factor was the scarcity of associated digital information as attributes in each unit, such as in the case of water retention capacity for which only a small part (40 units out of 800) could be related.

Yield information on coffee genotypes evaluated in the ESs and related to bioclimatic indices, other variables of interest related to vegetative growth, flowering, and quality, and molecular markers should be included in new research. Research should not be limited to the ESs, but should have wider national application, incorporating new research sites that this study identified as having potential strategic importance and therefore as being worthy of inclusion in the FNC's investigation plan.

## Conclusions

The coffee-growing regions in Colombia, based on bioclimatic indicators, can be classified into 12 large zones in which the coffee tree's responses are conditioned by the constraints or suitabilities of the environment, soils, and management. This information is valuable to the Colombian National Coffee Federation to guide their research and extension and will benefit the farmers of Colombia. The methodology and approach developed here can be used in other coffee-growing countries across the world.
